# Resignations and Appointments in Medical Schools

**Published:** 1858-07

**Authors:** 


RESIGNATIONS AND APPOINTMENTS IN MEDICAL SCHOOLS.—Dr. J. C. Nott, Professor of Anatomy in the University of Louisiana, has resigned the chair which he so ably filled last winter, and has been succeeded by Prof. T. G. Richardson, thus rendering vacant the Chair of Anatomy in the Pennsylvania College.

Professor Austin Flint has accepted the Chair of Clinical Medicine in the New Orleans School of Medicine. He will, however, retain his residence at Buffalo.

Dr. Sanford B. Hunt, in consequence of his retirement from the profession, has resigned the Professorship of Anatomy in the University of Buffalo, in which he is succeeded by Dr. Nichols, of that city.

Dr. J. C. Chisholm has been appointed to the Chair of Surgery in the Medical College of South Carolina. rendered vacant by the retirement of Professor E. Geddings. The Chair of Practice in the same institution, vacated by Professor Dickson, has been filled by the appointment of Dr. P. C. Gaillard.

Dr. S. G. Armor, of Dayton, Ohio, one of the most able and elegant lecturers in the Great West, has resigned the Professorship of Pathology and Clinical Medicine in the Missouri University.

